# Monitoring of the Complement System Status in Patients With B-Cell Malignancies Treated With Rituximab

**DOI:** 10.3389/fimmu.2020.584509

**Published:** 2020-11-19

**Authors:** Anna Felberg, Michał Taszner, Aleksandra Urban, Alan Majeranowski, Kinga Jaskuła, Aleksandra Jurkiewicz, Grzegorz Stasiłojć, Anna M. Blom, Jan M. Zaucha, Marcin Okrój

**Affiliations:** ^1^ Department of Cell Biology and Immunology, Intercollegiate Faculty of Biotechnology, University of Gdańsk and Medical University of Gdańsk, Gdańsk, Poland; ^2^ Department of Hematology and Transplantology, Medical University of Gdańsk, Gdańsk, Poland; ^3^ Department of Translational Medicine, Lund University, Malmö, Sweden

**Keywords:** obinutuzumab (GA101), non-Hodgkin’s lymphoma, complement system, chronic lymphocytic leukemia, rituximab

## Abstract

Rituximab is a pioneering anti-CD20 monoclonal antibody that became the first-line drug used in immunotherapy of B-cell malignancies over the last twenty years. Rituximab activates the complement system *in vitro*, but there is an ongoing debate on the exact role of this effector mechanism in therapeutic effect. Results of both *in vitro* and *in vivo* studies are model-dependent and preclude clear clinical conclusions. Additional confounding factors like complement inhibition by tumor cells, loss of target antigen and complement depletion due to excessively applied immunotherapeutics, intrapersonal variability in the concentration of main complement components and differences in tumor burden all suggest that a personalized approach is the best strategy for optimization of rituximab dosage and therapeutic schedule. Herein we critically review the existing knowledge in support of such concept and present original data on markers of complement activation, complement consumption, and rituximab accumulation in plasma of patients with chronic lymphocytic leukemia (CLL) and non-Hodgkin’s lymphomas (NHL). The increase of markers such as C4d and terminal complement complex (TCC) suggest the strongest complement activation after the first administration of rituximab, but not indicative of clinical outcome in patients receiving rituximab in combination with chemotherapy. Both ELISA and complement-dependent cytotoxicity (CDC) functional assay showed that a substantial number of patients accumulate rituximab to the extent that consecutive infusions do not improve the cytotoxic capacity of their sera. Our data suggest that individual assessment of CDC activity and rituximab concentration in plasma may support clinicians’ decisions on further drug infusions, or instead prescribing a therapy with anti-CD20 antibodies like obinutuzumab that more efficiently activate effector mechanisms other than complement.

## Introduction

CD20, a surface molecule present on most developmental stages of B lymphocytes, fulfills many conditions attributable to being a promising target for immunotherapy (1–5). The first anti-CD20 immunotherapeutic rituximab was clinically approved in 1997 (6). It became the first-line drug (usually in combination with chemotherapy), which significantly improved the survival of patients suffering from B cell leukemias and lymphomas (7, 8). Rituximab contains a human IgG1 Fc portion capable of activating immune effector mechanisms in man and rodents, including activation of the complement system and complement-dependent cytotoxicity (CDC) next to antibody-dependent cellular cytotoxicity (ADCC) and phagocytosis mediated by either Fc—or complement receptors (2, 9). On the other hand, immune escape and modulation of immune response by tumor cells and supracellular factors like the number of tumor cells and bioavailability of the drug influence the effectiveness of cancer eradication. Accordingly, indications that many patients are refractory to rituximab (10) reasoned the studies on the pivotal effector and resistance mechanisms, which often brought contradictory results. Our goal was to form coherent conclusions in the light of published data, with an emphasis on the role of the complement system. We also supplement these conclusions with original data showing the status of the complement system and the retention of the drug in patients with B cell malignancies receiving rituximab. In our opinion, monitoring of such parameters contributes to a personalized therapeutic approach highly appreciated in patients undergoing treatment with anti-CD20 antibodies.

### An Interplay Between Effector Mechanisms of Rituximab

Based on predominant effector mechanisms, anti-CD20 mAbs are classified into type I and type II antibodies (1, 3). Type I specimens are potent complement activators in contrast to type II, which directly exert cell death upon binding to the target cell. There are reports on limited rituximab-induced cell death in certain tumor B cell lines (11), nonetheless, rituximab is more efficient in the complement-mediated killing and categorized as a representative of type I. Notably, both type I and type II anti-CD20 mAbs can support ADCC induced by the binding of the Fc portion of antibody to Fc receptors localized on effector cells (predominantly NK cells). ADCC and CDC mechanisms may compete with each other as complement activation on the platform of cell-bound rituximab imposes the occupation of its Fc portion and results in a steric hindrance for the interaction with Fc*γ*RIII. This phenomenon was proven for the first time *in vitro* by Wang et al., who noticed that normal human serum or C5-depleted serum but not heat-inactivated serum, C1- and C3-depleted serum blocks NK cell activation (12). Further experiments in a syngeneic murine lymphoma model showed that complement depletion by application of cobra venom factor (CVF) before mAb administration resulted in longer survival than the application of mAb alone, thus suggesting that the ADCC mechanism is pivotal and complement activation is detrimental for the therapeutic effect of type I mAbs (13). However, one limitation of this and many other syngeneic mouse models is the usage of anti-CD20 other than rituximab whereas even subtle differences in target epitope or Ig structure outside of CDR regions may be critical for type I/II characteristics (14). A few studies analyzed effector mechanisms of type I anti-CD20 antibodies in transgenic mice expressing human CD20 (15–17). Beers et al. reported a dispensable role of the complement system in the elimination of CD20-positive cells by rituximab converted to mouse IgG2a isotype (equally efficient in CDC as the original rituximab) (16). Results of Tipton and colleagues suggest that antibody-mediated phagocytosis is the crucial effector mechanism (17) whereas Gong et al. showed that effective depletion of B cells may need different effectors depending on their location. Complement was found crucial for the elimination of B cells from the marginal zone in the spleen but not important in other sites (15). The other limitation in the context of the translational potential of *in vivo* studies in mouse models is the fact that mouse complement is very weak compared to other mammals (18, 19), and therefore experiments performed in the mouse model introduce the risk of under-appreciation of CDC as an effector mechanism. Nonetheless, there is a number of the mouse *in vivo* studies that either support (20–22) or question (16, 17, 23, 24) the critical role of complement in the therapeutic effect of rituximab. There is a lack of conclusive *in vivo* studies performed in animal models with complement activity comparable to humans (e.g., rat, guinea pig, and dog). A single study in nude rats with intracerebral lymphoma xenograft successively treated with rituximab suggests complement involvement (25). However, a separate and more detailed investigation must ensure the extrapolation of this conclusion.

Observations from clinics and *ex vivo* experiments in man also bring ambiguous conclusions. ADCC reactions may play a role in the therapeutic effect of rituximab as a low number of NK cells correlated with poor clinical outcome (26). A higher response rate to rituximab and higher progression-free survival of patients with follicular lymphoma was shown in individuals with a polymorphism in Fc*γ*RIIIa (CD16), which renders a high affinity to IgG1 (27, 28) but these findings were not confirmed in a larger clinical study (29). Additionally, clinical response and duration of response to rituximab were correlated with polymorphism of the C1qA gene that associates with low levels of C1q—the first component of the classical complement pathway (30). Contrarily, addition of fresh frozen plasma to CLL patients markedly improved their clinical outcome, even when previous administrations of rituximab were ineffective (31, 32). These data suggest that the CDC/ADCC interplay depends either on model or supracellular factors like the number of tumor cells and the expression of the target antigen. Since the threshold necessary for effective ADCC is lower than that for CDC (33), these two competitive effector mechanisms may act cooperatively, i.e. in case of a heterogeneous population of tumor cells, ADCC eliminates these of low CD20 expression whereas complement eradicates cells with high CD20 content. The number of tumor cells is another parameter important in the context of rituximab’s effector mechanisms. Boross et al. showed that injection of rituximab to FcR*γ*-deficient mice was ineffective at a high load of tumor cells and that, in contrast to a challenge with a low number of tumor cells, effective elimination demands the cooperation of complement and ADCC and the presence of functional complement receptor 3 (CR3) on blood phagocytes (34). The role of receptors for complement-derived opsonins is also underlined by Lee at al., who developed rituximab RA801 mutant non-bondable to human or mouse Fc receptors but retaining complement activation potential (35). While PBMC and PMN were not able to eliminate RA801-opsonized CD20-positive cells *ex vivo* without the addition of serum depleted of the C9 component, there was no difference in human CD20-positive Ramos cells’ eradication in *in vivo* nude mouse model between original rituximab and RA801 mutant (35). Yet, eradication of mouse EL4 lymphoma cells expressing human CD20 by rituximab, but not RA801, was impaired in mice additionally lacking all Fc receptors. This can be explained by the higher CDC efficacy of RA801 (4.5-fold lower CH_50_ value) compared to rituximab. Nonetheless, such results underline two important issues: i) extrapolation of conclusions obtained from the studies on one mAb to the other, even closely related mAb, is not reliable, and ii) the relative importance of rituximab’s effector mechanisms heavily depends on the target cells. Therefore the seemingly contradictory results showing successful depletion of B cells by rituximab-like antibodies in mice with functional macrophages and Fc*γ* receptor-dependent pathways but lacking functional complement or ADCC mechanism (16, 23, 36) should not be surprising.

### Rituximab (Type I) or Type II Anti-CD20 Immunotherapeutics?

Since both type I and type II anti-CD20 antibodies are nowadays available in clinics, a relevant dilemma is which of these two types is superior for particular patients. Complicated interplay between effector mechanisms and heterogeneity of targets in B cell malignancies in conjunction with supracellular factors make a unanimous answer problematic. Due to the same reason, the role of the complement system in the therapeutic effect cannot be generally ruled out or confirmed. However, assuming that under certain circumstances patients may benefit from complement activation by rituximab, parallel monitoring of the complement system parameters enables selecting subjects with functional impairment, saturation, or unresponsiveness of this effector mechanism, who may benefit more from type II antibodies, e.g., obinutuzumab that more efficiently activates effectors other than complement (37). Another parameter deserving control in case of usage of type I anti-CD20 antibodies is their retention in blood. When excessively administered, they may lead to loss of target antigen *via* internalization (3) and trogocytic removal (38, 39). Conversely, administering type II antibodies results in higher stability of surface CD20 antigen (40). In experimental models, the saturation of the CDC takes place much faster than the saturation of C3b deposition on target cells, thus overdosing provokes exhaustion of the complement system (41, 42). Such exhaustion affects mostly the initial components of the classical pathway, namely, C1 and C2, which are present in serum at much lower molar concentrations than C3 and act as a bottleneck of the whole pathway. Since malignant B cells are typically equipped with a set of complement inhibitors that affect C3/C5 convertases (43, 44), their activity will also lead to the consumption of downstream components C1 and C2. Therefore, too high concentration of rituximab and potent intrinsic complement inhibition by tumor cells may not only dampen CDC at consecutive infusions of the drug but also lead to the selection of tumor cells with low expression of CD20 antigen. Transient loss of CD20 on tumor B cells following rituximab infusion was observed in CLL patients and considered as one of the causes of the limited efficacy of antitumor mAbs (42, 45).

Previously we proposed a calcein release assay on Raji cells as a method for monitoring CDC potential of serum collected from patients treated with type I anti-CD20 antibodies (46). There are several advantages of this method over the routinely used CH_50_ assay performed on sensitized sheep erythrocytes: i) usage of human tumor cells bearing both molecular target (CD20) for dedicated immunotherapeutics and human complement inhibitors (CD46, CD55, CD59) (43) fully compatible with human complement, ii) adequate sensitivity of target cells to complement-mediated lysis and iii) lower inter-assay variability compared to CH_50_ assay (46). Using this approach, we measured the CDC potential of serum samples collected before and after each infusion of rituximab in 17 patients with various B cell malignancies. In another version of these experiments, we supplemented the analyzed sera with saturating concentration of rituximab to evaluate whether putative post-infusion complement depression overlapped with consecutive infusions. In parallel, we measured rituximab concentrations in each sample. The combined results of these experiments reveal the net functional effect of rituximab retention and individual competence of the complement system, which altogether may support the clinician’s decision on modification of the therapeutic schedule or switch into type II anti-CD20 antibodies.

## Methods

### Patients and Treatment

All samples collected from patients and healthy volunteers were obtained after written informed consent, in accordance with the Declaration of Helsinki and with approval from The Local Bioethical Committee at Medical University of Gdańsk (approval number: NKBBN/500/2016). The cohort consisted of 17 patients admitted to Dept. of Hematology and Transplantology of Medical University of Gdańsk, 7 of which were diagnosed with CLL and 10 with different forms of NHL. All patients had no prior therapies. They were administered with 375 mg/m2 rituximab over the period from 2 to 5 h in four-week intervals for 4 to 8 cycles. All but two patients received concomitant chemotherapy. Detailed patients’ characteristics are given in **Table 1**. Response to treatment was assessed according to iwCLL guidelines (47). Blood drawn immediately before and after rituximab infusions was used for serum preparation, as described in (48) and for preparation of EDTA-plasma.

**Table 1:** Patients’ characteristics.

Patient #	Diagnosis	Combinedchemotherapy	Clinical response (the way of assessment)	Lymphocyte count before infusions 1-4 (CLL patients only) [10^9^/ml]			
				1^st^	2^nd^	3^rd^	4^th^
1	DLBCL	CHOP	mCR (PET)				
6	HGL	EPOCH	PROG (PET)				
8	PMBCL	EPOCH	mCR (PET)				
9	DLBCL	CHOP	PROG (CT)				
10	BL	codox/ivac	mCR (PET)				
11	FL	COP	PR (CT)				
12	MZL	none	mCR (PET)				
17	CLL	none	PR (clinical)	91.44	42.79	15.03	9.44
18	CLL	FC	CR (clinical)	46.16	3.42	1.73	0.75
19	HGL	CHOP	PROG (CT)				
20	MZL	COP/bendamustine	PR (CT)				
21	CLL	FC	CR (clinical)	64.76	0.83	0.23	0.34
23	CLL	FC	CR (clinical)	128.0	4.37	2.58	0.85
26	CLL	FC	CR (clinical)	81.63	0.48	0.56	0.32
27	CLL	FC	PR (clinical)	90.96	2.29	2.63	0.93
31	FL	COP	CR (CT)				
33	CLL	FC	CR (MRD -)	6.67	2.82	0.17	1.05

Diagnosis: DLBCL, diffused large B cell lymphoma; HGL, high grade lymphoma; PMBCL, primary mediastinal B cell lymphoma; FL, follicular lymphoma; BL, Burkitt lymphoma; MZL, marginal zone B cell lymphoma; CLL, chronic lymphocytic leukemia.

Chemotherapy: CHOP, cyclophosphamide + hydroxydaunorubicin + oncovin + prednisone; EPOCH, etoposide + prednisone + oncovin + cyclophosphamide + hydroxydaunorubicin; COP, cyclophosphamide + oncovin + prednisone; FC, fludarbine + cyclophosphamide.

Clinical response: CR, complete response; mCR, metabolic clinical response; PR, partial response, PROG, progression; PET, positron emission tomography; CT, computed tomography; MRD, minimal residual disease.

### Sample Handling

After collection and preparation, which was accomplished in approximately 30 min after blood collection, serum and plasma samples were aliquoted and kept at −80°C until the time of the experiment. Repetitive freezing and thawing were avoided, and the same rule was applied to normal human serum (NHS) and normal human plasma (NHP), which were prepared from the blood of healthy volunteers and pooled. NHS was then used as a positive control in the CDC assay. NHP was used as a milieu for the preparation of the calibration curve in ELISA-based measurements of C4d and TCC. Heat-inactivated normal human serum (Δ NHS) was prepared from NHS heated to 56°C for 30 min and then cleared by centrifugation at 3000 x G for 5 min. Δ NHS was used as a negative control in CDC assays as heat-inactivation depletes complement activity. Working dilutions of serum and plasma were prepared only before experiments in chilled tubes or microplates kept on ice.

### Cell Lines

Raji, Ramos, Namalwa, SU-DHL-4 cells were obtained from the American Type Culture Collection. Cells were aliquoted and cryopreserved after the first few passages. Cells used for experiments were grown from such stock aliquots in RPMI 1640 medium with l-glutamine (ATCC) supplemented with 10% fetal bovine serum (ATCC) at 37 °C and humidified 5% CO_2_ atmosphere. Cells were routinely checked for Mycoplasma contamination by DAPI staining (49) when cultured and never kept in continuous culture for more than 10 passages. The primary culture of CLL cells was established from heparinized patients’ blood. Lymphocyte fraction was isolated using Lymphoprep (Stemcell Technologies) according to the manufacturer’s instruction and assessed as a homogenous population by flow cytometry (>98% of gated objects) showing CD20 expression. Then CLL cells were cultured in a 1:1 mixture of RPMI 1640: DMEM (HyCult) medium supplemented with 10% FBS.

### Assessment of Rituximab Concentrations

Rituximab concentration in samples collected just before and just after each infusion was measured using an enzyme-linked immunosorbent assay. 96-well ELISA MaxiSorp plates (ThermoFisher Scientific) were coated with 1 µg/ml of anti-rituximab (anti-idiotype) antibody RB01 (R&D Systems) and blocked with washing buffer (50 mM Tris-HCl, 0.15 M NaCl, 0.1% Tween, pH 7.5) supplemented with 3% fish skin gelatin (Sigma-Aldrich). Patients’ serum was diluted to the final concentration of 0,125% in PBS with 0.02% Tween-20 and 0.02M EDTA. Rituximab (Roche) serially diluted in NHS was used for the preparation of the calibration curve. The horseradish peroxidase-conjugated goat anti-mouse antibody (Dako, P0447) was used for detection. The assay was developed using 3,3′,5,5′-Tetramethylbenzidine (TMB) (Sigma-Aldrich), and absorbance readout at 450 nm was measured using a Synergy H1 microplate reader (Biotek).

### CDC Assay

Complement-dependent cytotoxicity (CDC) functional assay was performed as described in (46). Briefly, cells previously loaded with calcein-AM (Sigma) were pelleted onto V-shape microplate wells and overlaid with 50 μl of the indicated serum with or without addition of rituximab. After 30 min of incubation fluorescence of calcein released into the supernatant was measured at 490 nm/520 nm excitation/emission wavelength in Synergy H1 microplate reader (Biotek).

### Measurement of Complement Activation Markers

Measurements of the early activation marker of the classical complement pathway, C4d, and the marker of terminal complement pathway activation TCC were performed as described in (50), with slight modifications regarding the TCC sandwich ELISA assay. Instead of zymosan-activated serum, serial dilutions of purified sC5b-9 complex (Complement Technology) in 5% NHP solution in PBS with 0.02% Tween-20 and 0.02M EDTA were used for calibration curve. Detection was achieved using polyclonal rabbit anti-human sC5b-9 neo antibody (Complement Technology) followed by horseradish peroxidase-conjugated goat anti-rabbit antibody (Dako).

### Statistics

The grouped analyses of differences in CDC potential and concentration of complement activation markers between pre- and post-infusion serum samples collected at each infusion were performed by multiple Sidak’s comparison tests. Calculations were supported by GraphPad 6 software (Prism).

## Results

**Figure 1:**
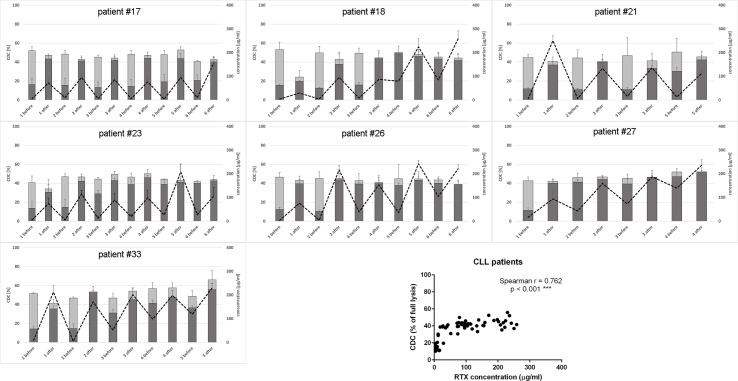
CDC potential and rituximab concentration in serum samples collected from CLL patients. CDC potential was assessed in calcein release assay performed using Raji cells incubated with 10% patient’s serum. Dark bars represent CDC levels of patients’ sera non-supplemented with extra rituximab, grey bars represent CDC levels when sera were supplemented with 50 µg/ml of rituximab. Dotted line represents rituximab concentration (right Y axis). Each serum was tested in three independent experiments, error bars indicate standard deviation.

**Figure 2:**
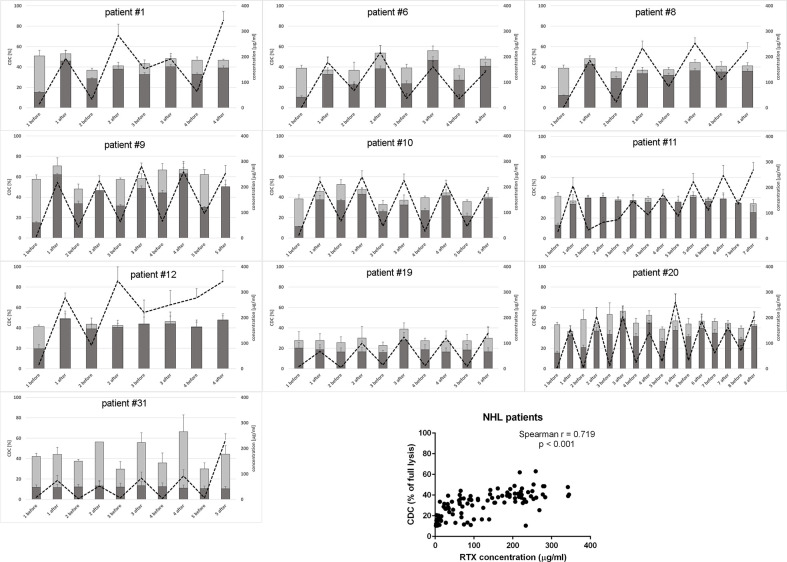
CDC potential and rituximab concentration in serum samples collected from NHL patients. CDC potential was assessed in calcein release assay performed using Raji cells incubated with 10% patient’s serum. Dark bars represent CDC level of patients’ sera non-supplemented with extra rituximab, grey bars represent CDC level when sera were supplemented with 50 µg/ml of rituximab. Dotted line represents rituximab concentration (right Y axis). Each serum was tested in three independent experiments, error bars indicate standard deviation.

We analyzed the CDC activity of patients’ sera collected immediately before and after each infusion of rituximab in two different experimental settings: i) without the addition of a new dose of rituximab and ii) with saturating concentration of rituximab added to patients’ serum. The first measurement aimed to assess the cytotoxic activity of serum during the treatment, which reflected the retention of rituximab and the competence of the complement system. The second measurement was performed upon conditions, which imposed complement activity but not rituximab concentration, as a CDC-limiting factor. Thus, the latter assessed the immediate post-infusion complement depletion and whether such putative depletion overlapped with the next infusion. The results obtained for CLL and NHL patients are shown in **Figures 1** and **2**, respectively.

Only one CLL patient (#18) showed spectacular, significantly lower CDC of rituximab-supplemented post-infusion serum sample compared to the analogical pre-infusion sample and such CDC depletion was only observed at the first infusion (**Figure 1** and **Supplementary Statistics File**). Out of seven CLL patients included in the study, four accumulated more than 100 mg/ml of rituximab before infusion 3 (patient #27), infusion 4 (patients #18 and #33), and infusion 6 (patient #26). Similarly, six out of ten patients with NHL accumulated rituximab at the level of 100 mg/ml before infusion 2 (patient #12), infusion 3 (#1, #8, and #11) infusion 5 (#9) and infusion 8 (#20), respectively (**Figure 2**). In both groups of patients, there was a significant correlation between rituximab concentration and CDC exerted on Raji cells, with the saturation level of CDC achieved at rituximab concentration around 50 mg/ml (inlets in **Figures 1** and **2**). We found one NHL patient (#19) who presented depressed CDC throughout all infusions, even when serum samples were supplemented with extra rituximab (**Figure 2**). Interestingly, this patient did not respond to the therapy. One NHL patient (#31) exhibited a low level of CDC in both pre- and post-infusion serum samples, but all his samples regained functionality when supplemented with extra rituximab. Nonetheless, patient #31 achieved a complete response to the treatment.

**Figure 3:**
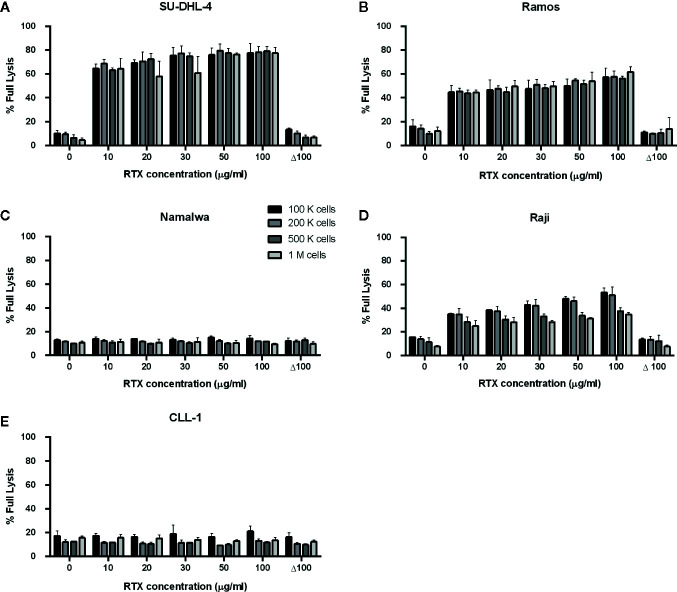
CDC exerted in 50% normal human serum by rituximab at concentration range 10-100 µg/ml. CDC was examined on four CD20-positive cell lines: SU-DHL-4 **(A)**, Ramos **(B)**, Namalwa **(C)**, Raji **(D)** and fresh culture of CLL cells **(E)**. Supernatant collected from calcein-labelled cells lysed with 30% DMSO diluted in PBS served as the indicator of 100% (full) lysis. Readout obtained for heat-inactivated serum (Δ NHS) served as negative control, i.e. background lysis independent on complement activation. Cells were tested at quantities 1, 2, 5, and 10 × 10^5^ cells/50 µl, Δ 100 group represents heat-inactivated normal human serum supplemented with 100 µg/ml of rituximab. Data were collected from three independent experiments, error bars indicate standard deviation.

Previously we characterized Raji as a cell line moderately sensitive to CDC exerted by anti-CD20 mAbs. Incubation of Raji cells in 50% NHS supplemented with CDC-saturating concentration of rituximab (50 µg/ml) yielded in c.a. 50% of lysis (43). In the current experiments performed in 10% of patients’ sera (**Figures 1** and **2**), we observed the highest impact of rituximab on the CDC in a concentration range from 10 to 100 µg/ml. Therefore we attempted to assess the effect of the same concentration range either at the different load of tumor cells or on other CD20-positive tumor cells of different sensitivity to CDC (**Figure 3**). Experiments performed in 50% NHS should demonstrate the highest CDC effect theoretically attainable in blood. Raji cells showed CDC increase from 35% to 53% at 100.000 cells and from 25 to 35% at 1M cells when rituximab concentration increased from 10 to 100 µg/ml. Ramos cells showed increased CDC from 44 to 61% of full lysis but there was no effect of increased cell number. Similarly, a 10-fold increase of cell number did not significantly affect the lysis of SU-DHL-4 cells, where the CDC oscillated from 65% at 10 µg/ml of rituximab to 77% at 100 µg/ml of rituximab. Rituximab was ineffective in the killing of Namalwa cells and fresh culture of CLL cells, irrespectively on concentration (**Figure 3**).

**Figure 4:**
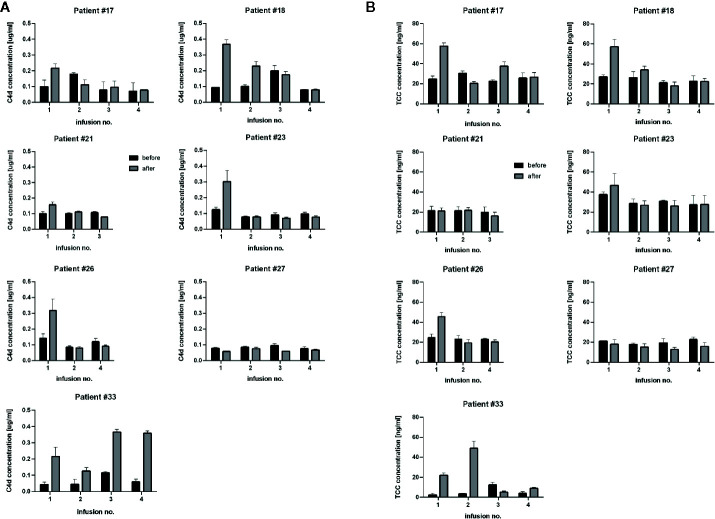
Determination of levels of C4d and TCC, complement activation markers. Graphs show C4d concentration **(A)** and TCC concentration **(B)** in sera collected before (black bars) and after (grey bars) consecutive rituximab infusions in CLL patients. Data were collected from three independent measurements, error bars represent standard deviation.

CLL patients possess tumor cells circulating in their bloodstream, which are much better accessible for effector mechanisms than tumor cells residing in bone marrow, lymph nodes or other extravascular locations. We analysed appearance of complement activation markers in plasma samples from the CLL patients during the first four infusions (when available). Significant increase of either C4d and TCC were observed (if any) mainly after the first infusions (**Figure 4**), corresponding with the high number of circulating tumor cells further eliminated during the treatment (see **Table 1**). However, patients #21 and #27 did not show signs of strong systemic complement activation, despite the ability of their sera to exert CDC *in vitro* (**Figure 1**). Importantly, levels of both C4d and TCC markers do not correlate with CDC exerted on different target cells (**Supplementary Figure 1**) and should be considered as qualitative rather than quantitative measures of CDC *in vivo*.

## Discussion

There is no unanimous opinion on the role of complement in the therapeutic effect of type I anti-CD20 antibodies. Results of *in vivo* animal studies seem to be model-dependent (reviewed in (4)), and the predictive value of *ex vivo* CDC assays in uncertain. Bordon et al. reported the vulnerability of isolated CD20-positive tumor cells to the CDC as a predictor of clinical response to rituximab (51), but two other studies presented contradictory results (52, 53). A strong argument for the complement role in CLL immunotherapy is the observation that clinical response to rituximab improved after supplementation with fresh-frozen plasma (31, 32). On the other hand, up to 40% of CLL patients may have deficiencies or low levels of circulating complement proteins (54). Therefore the first question we asked in the current study is whether the CDC activity of sera collected from the patients receiving rituximab is sufficient to lyse a model CD20-positive Raji cells. The functional assay we performed to answer this question is much more informative than measurements of the main complement components, whose physiological concentration range varies substantially (e.g., 0.6–1.4 g/L for C3 and 0.1–0.33 g/L for C4) (55). Notably, even C3 concentration as low as 0.18 g/L was reported sufficient to maintain a proper complement function (56).

Raji cell line is characterized as a moderately sensitive to rituximab compared to other B-cell lymphomas, thus enabling observation of either depressed or higher than average complement activity in CDC assays (43). Previously we demonstrated the utility of this model for the mirroring of the anti-CD20 antibody-driven complement consumption and found superior sensitivity of the assay when 10% instead of 50% serum was used (46). Importantly, 10% serum is a surrogate of the complement content in lymph or extravascular fluids, a natural microenvironment of lymphoma (13, 57, 58). However, an increase of NHS concentration from 10% to 50% did not result in a significant increase of CDC in Raji cells, as demonstrated in (43). In the current study, the readout of CDC assay at 10% patients’ serum that contained saturating concentration of rituximab (**Figures 1** and **2**) in most of the cases was not significantly different from the readout obtained at 50% NHS (see **Figure 3D**, bar for the concentration of 50 µg/ml and 100 k cells). Nonetheless, there were few exceptions from this rule. All post-infusion samples of patients #19 and #31 had low CDC activity. Supplementation with additional 50 µg/ml of rituximab markedly improved the CDC readout in patient #31.

We did not study the complement activity of serum over several hours after infusion as others did (41, 59) but found only one patient (#18) who showed signs of complement exhaustion immediately after infusion. Importantly, such exhaustion did not overlap with the next infusion indicating that a four-week interval is enough for the restoration of the complement pool. These results are in agreement with another study, which analyzed the effect of ofatumumab, a stronger CDC-activating anti-CD20 antibody (60), applied in a 2-week interval (46).

CDC potential of pre-infusion serum samples (without addition of rituximab) correlated with the amount of accumulated rituximab in both CLL and NHL patients (inlets in **Figures 1** and **2**). The study by Berinstein et al. evaluated pharmacokinetics of rituximab in 137 non-Hodgkin’s lymphoma patients, who received the 375 mg/m^2^ dose once weekly for four injections (61). The median difference in rituximab concentration between post- and pre-infusion serum was approx. 250-270 µg/ml, whereas the median level of rituximab in pre-infusion samples was 63 µg/ml, 124 µg/ml, and 186 µg/ml at second, third, and fourth administration, respectively. Significantly higher accumulation of rituximab was noticed in responders to the therapy before the second and fourth infusion. Accumulation of the drug may be explained by a decreased number of accessible tumor cells in responders, but further studies also suggest the loss of target antigen due to internalization and trogocytic removal as a possible explanation (3, 62–64). In our cohort, the differences between post- and pre-infusion levels of serum rituximab were from 25 to 246 µg/ml in CLL patients and from 30 to 279 µg/ml in NHL patients. NHL patients who gradually accumulated rituximab throughout all infusions achieved complete response (#1, #8, and #12), partial response (#11, #20) or progression (#9). Part of the NHL patients with no gradual accumulation of rituximab had progressive disease (#6, #19), but the other part (#10 and #31) showed complete response, so there was no clear segregation into responders and non-responders in terms of rituximab accumulation. These results, opposite to the previous study, can be explained by a four-week instead of one-week interval in rituximab dosing. However, our results show that even at a four-week interval, there are patients (#1, #8, #11, and #12), which accumulate the amounts rituximab comparable to these delivered at the first infusion. Excessively administrated rituximab provides a risk for the selection of a CD20-low population of tumor cells (62). On the other hand, the bioavailability of rituximab in lymph nodes and other extravascular sites is lower than in serum (65). Such a high accumulation of rituximab and concomitant saturation of CDC potential in pre-infusion sera imposes a question if the additional dosing is necessary or counterproductive. Thus, a biopsy of lymphoma cells stained for either cell-bound rituximab or free antigenic CD20 sites will give a valuable hint on whether the therapeutic schedule should be modified or the therapy should be changed to type-II anti-CD20 antibodies such as obinutuzumab, which is superior for the killing of tumor cells *via* ADCC and direct mechanisms (37).

Circulating CD20-positive cells in CLL patients are much more accessible for complement than NHL cells in extravascular locations. Therefore, complement activation by rituximab on circulating CLL cells should be immediately mirrored by the appearance of complement activation markers such as C4d and TCC. C4d is a marker of early stages of the classical complement pathway activation, which leads to opsonization (and complement-dependent phagocytosis) and anaphylaxis. Soluble TCC is formed upon assembly of membrane attack complex (MAC) and indicates CDC. Previously we validated C4d and TCC assays on the cohort of 31 CLL patients and found that increase of TCC in the post-infusion samples took place when an increase of C4d was also observed (50). Nonetheless, the formation of C4d and TCC must depend on the expression level of either CD20 or complement inhibitors present on tumor cells and in patients’ sera. We characterized numerous CD20-positive cell lines (including these presented in **Supplementary Figure 1**) and fresh CLL cultures for their expression of CD20 and endogenous complement inhibitors (43). As substantial differences were found in these cells, we assume similar variability in patients. Therefore, the concentration of detected markers cannot be directly associated with CDC intensity, as shown in **Supplementary Figure 1**, and directly compared between individuals. The appearance of C4d and TCC markers indicates whether the complement activation took place and whether it proceeded up to the terminal stages, respectively. The highest increase of complement activation markers should be expected after the first infusion when a high number of CD20-positive tumor cells is present. Indeed, most CLL patients had increased levels of C4d and TCC after the first infusion with a tendency to flatten the differences at consecutive infusions. Except for patient #17, who received rituximab as monotherapy and except for patient #33, the drop in absolute lymphocyte count after the first rituximab infusion in CLL patients was greater than 90% (**Table 1**). Patient #17 achieved a partial response and showed neither gradual accumulation of rituximab nor saturation of CDC serum activity in any of the pre-infusion samples (**Figure 2**). Two CLL patients showed a marginal (#21) or no increase (#27) in C4d. Accordingly, both patients showed no increase in TCC (**Figure 4**). Notably, patient #21 achieved a complete response, unlike patient #27, who responded partially and showed accumulated rituximab throughout all infusions and saturated serum CDC potential already before the second infusion (**Figure 1**).

Our analyses of the complement system competence accompanied by the measurements of rituximab concentration in serum during consecutive infusions performed in the group of 17 patients with heterologous B-cell malignancies are not sufficient to answer the question about the role of complement in the therapeutic effect of rituximab. However, there are two important observations from our study. Irrespectively of serum and drug concentration, rituximab could not exert CDC in freshly isolated CLL cultures (**Figure 3E**) and in Namalwa cells (**Figure 3C**), which express the relative levels of CD20 and complement inhibitors comparable to these observed in CLL cultures (33, 40, 43). These results are in line with our previous publication showing the inability of rituximab to lyse CLL cells isolated from six patients (43). We conclude that CDC cannot be a sole killing mechanism of CLL cells *in vivo* when rituximab is applied as a monotherapy (as in patient #17), however, concomitant chemotherapy may additionally sensitize tumor cells for CDC, and complement receptor-driven phagocytosis cannot be ruled out. The second issue worth underlining is the fact that even in such a small group of heterologous patients treated with a standard rituximab dose, there were examples of individuals, who deserved a personalized approach. These examples were patient #27 who accumulated a high concentration of rituximab in serum and had fully functional complement but presented no increase of complement activation markers, patients #19 and #31 who had depressed or non-functional complement, and patients #1, #8, #11, and #12 who showed substantial accumulation of rituximab and additionally (#11 and #12) saturated CDC potential of their sera. Monitoring of the complement status and concentration of cell-free rituximab may suggest to clinicians that the ongoing therapy should be continued with type II anti-CD20 antibodies, impose the re-evaluation of a molecular target for the drug, or a delay of further infusions, respectively.

## Data Availability Statement

The original contributions presented in the study are included in the article/**Supplementary Material**. Further inquiries can be directed to the corresponding author.

## Ethics Statement

The studies involving human participants were reviewed and approved by The Local Bioethical Committee at Medical University of Gdańsk. The patients/participants provided their written informed consent to participate in this study.

## Author Contributions

AF, AU, KJ, AJ, and GS performed the experiments and/or optimized assays used in the study. MT, AM, and JZ diagnosed the patients and collected clinical material. AB, MO, and JZ wrote the manuscript. MO conceived the idea of the study. All authors contributed to the article and approved the submitted version.

## Funding

This work was supported by National Science Centre Poland grant no. 2014/14/E/NZ6/00182 and Cancerfonden.

## Conflict of Interest

The authors declare that the research was conducted in the absence of any commercial or financial relationships that could be construed as a potential conflict of interest.

## Abbreviations

ADCC, antibody-dependent cellular cytotoxicity; CDC, complement-dependent cytotoxicity; CLL, chronic lymphocytic leukemia; CR3, complement receptor 3; CVF, cobra venom factor; MAC, membrane attack complex; NHL, non-Hodgkin’s lymphoma; NHP, normal human plasma; NHS, normal human serum; TCC, terminal complement complex; Δ NHS, heat-inactivated normal human serum.

## References

[1] Winiarska M Glodkowska-Mrowka E Bil J Golab J Molecular mechanisms of the antitumor effects of anti-CD20 antibodies. Front Biosci (2010) 16:277–306. 10.2741/3688 21196171

[2] Glennie MJ French RR Cragg MS Taylor RP Mechanisms of killing by anti-CD20 monoclonal antibodies. Mol Immunol (2007) 44:3823–37. 10.1016/j.molimm.2007.06.151 17768100

[3] Beers SA French RR Chan HT Lim SH Jarrett TC Vidal RM Antigenic modulation limits the efficacy of anti-CD20 antibodies: implications for antibody selection. Blood (2010) 115:5191–201. 10.1182/blood-2010-01-263533 20223920

[4] Okroj M Osterborg A Blom AM Effector mechanisms of anti-CD20 monoclonal antibodies in B cell malignancies. Cancer Treat Rev (2013) 39:632–9. 10.1016/j.ctrv.2012.10.008 23219151

[5] Marshall MJE Stopforth RJ Cragg MS Therapeutic Antibodies: What Have We Learnt from Targeting CD20 and Where Are We Going? Front Immunol (2017) 8:1245. 10.3389/fimmu.2017.01245 29046676 PMC5632755

[6] Maloney DG Grillo-Lopez AJ White CA Bodkin D Schilder RJ Neidhart JA IDEC-C2B8 (Rituximab) anti-CD20 monoclonal antibody therapy in patients with relapsed low-grade non-Hodgkin’s lymphoma. Blood (1997) 90:2188–95. 10.1182/blood.V90.6.2188.2188_2188_2195 9310469

[7] Murawski N Pfreundschuh M New drugs for aggressive B-cell and T-cell lymphomas. Lancet Oncol (2010) 11:1074–85. 10.1016/S1470-2045(10)70210-2 21051020

[8] Marcus R Davies A Ando K Klapper W Opat S Owen C Obinutuzumab for the First-Line Treatment of Follicular Lymphoma. N Engl J Med (2017) 377:1331–44. 10.1056/NEJMoa1614598 28976863

[9] Harjunpaa A Junnikkala S Meri S Rituximab (anti-CD20) therapy of B-cell lymphomas: direct complement killing is superior to cellular effector mechanisms. Scand J Immunol (2000) 51:634–41. 10.1046/j.1365-3083.2000.00745.x 10849376

[10] Solal-Celigny P Leconte P Bardet A Hernandez J Troussard X A retrospective study on the management of patients with rituximab refractory follicular lymphoma. Br J Haematol (2018) 180:217–23. 10.1111/bjh.15023 29230799

[11] Awasthi A Ayello J Van de Ven C Elmacken M Sabulski A Barth MJ Obinutuzumab (GA101) compared to rituximab significantly enhances cell death and antibody-dependent cytotoxicity and improves overall survival against CD20(+) rituximab-sensitive/-resistant Burkitt lymphoma (BL) and precursor B-acute lymphoblastic leukaemia (pre-B-ALL): potential targeted therapy in patients with poor risk CD20(+) BL and pre-B-ALL. Br J Haematol (2015) 171:763–75. 10.1111/bjh.13764 26471982

[12] Wang SY Racila E Taylor RP Weiner GJ NK-cell activation and antibody-dependent cellular cytotoxicity induced by rituximab-coated target cells is inhibited by the C3b component of complement. Blood (2008) 111:1456–63. 10.1182/blood-2007-02-074716 PMC2214766 18024795

[13] Wang SY Veeramani S Racila E Cagley J Fritzinger DC Vogel CW Depletion of the C3 component of complement enhances the ability of rituximab-coated target cells to activate human NK cells and improves the efficacy of monoclonal antibody therapy in an in vivo model. Blood (2009) 114:5322–30. 10.1182/blood-2009-01-200469 PMC2796137 19805620

[14] Meyer S Evers M Jansen JHM Buijs J Broek B Reitsma SE New insights in Type I and II CD20 antibody mechanisms-of-action with a panel of novel CD20 antibodies. Br J Haematol (2018) 180:808–20. 10.1111/bjh.15132 29468712

[15] Gong Q Ou Q Ye S Lee WP Cornelius J Diehl L Importance of cellular microenvironment and circulatory dynamics in B cell immunotherapy. J Immunol (2005) 174:817–26. 10.4049/jimmunol.174.2.817 15634903

[16] Beers SA Chan CH James S French RR Attfield KE Brennan CM Type II (tositumomab) anti-CD20 monoclonal antibody out performs type I (rituximab-like) reagents in B-cell depletion regardless of complement activation. Blood (2008) 112:4170–7. 10.1182/blood-2008-04-149161 PMC2582008 18583569

[17] Tipton TR Roghanian A Oldham RJ Carter MJ Cox KL Mockridge CI Antigenic modulation limits the effector cell mechanisms employed by type I anti-CD20 monoclonal antibodies. Blood (2015) 125:1901–9. 10.1182/blood-2014-07-588376 25631769

[18] Ong GL Mattes MJ Mouse strains with typical mammalian levels of complement activity. J Immunol Methods (1989) 125:147–58. 10.1016/0022-1759(89)90088-4 2607149

[19] Bergman I Basse PH Barmada MA Griffin JA Cheung NK Comparison of in vitro antibody-targeted cytotoxicity using mouse, rat and human effectors. Cancer Immunol Immunother (2000) 49:259–66. 10.1007/s002620000120 PMC11036976 10941909

[20] Di Gaetano N Cittera E Nota R Vecchi A Grieco V Scanziani E Complement activation determines the therapeutic activity of rituximab in vivo. J Immunol (2003) 171:1581–7. 10.4049/jimmunol.171.3.1581 12874252

[21] Cragg MS Glennie MJ Antibody specificity controls in vivo effector mechanisms of anti-CD20 reagents. Blood (2004) 103:2738–43. 10.1182/blood-2003-06-2031 14551143

[22] Golay J Cittera E Di Gaetano N Manganini M Mosca M Nebuloni M The role of complement in the therapeutic activity of rituximab in a murine B lymphoma model homing in lymph nodes. Haematologica (2006) 91:176–83. 10.3324/%x 16461301

[23] Uchida J Hamaguchi Y Oliver JA Ravetch JV Poe JC Haas KM The innate mononuclear phagocyte network depletes B lymphocytes through Fc receptor-dependent mechanisms during anti-CD20 antibody immunotherapy. J Exp Med (2004) 199:1659–69. 10.1084/jem.20040119 PMC2212805 15210744

[24] Minard-Colin V Xiu Y Poe JC Horikawa M Magro CM Hamaguchi Y Lymphoma depletion during CD20 immunotherapy in mice is mediated by macrophage FcgammaRI, FcgammaRIII, and FcgammaRIV. Blood (2008) 112:1205–13. 10.1182/blood-2008-01-135160 PMC2515149 18495955

[25] Miyake Y Okoshi Y Machino T Chiba S Treatment of central nervous system lymphoma in rats with intraventricular rituximab and serum. Int J Hematol (2010) 92:474–80. 10.1007/s12185-010-0669-7 20820968

[26] He L Zhu HY Qin SC Li Y Miao Y Liang JH Low natural killer (NK) cell counts in peripheral blood adversely affect clinical outcome of patients with follicular lymphoma. Blood Cancer J (2016) 6:e457. 10.1038/bcj.2016.67 27518240 PMC5022180

[27] Cartron G Dacheux L Salles G Solal-Celigny P Bardos P Colombat P Therapeutic activity of humanized anti-CD20 monoclonal antibody and polymorphism in IgG Fc receptor FcgammaRIIIa gene. Blood (2002) 99:754–8. 10.1182/blood.v99.3.754 11806974

[28] Weng WK Levy R Two immunoglobulin G fragment C receptor polymorphisms independently predict response to rituximab in patients with follicular lymphoma. J Clin Oncol (2003) 21:3940–7. 10.1200/JCO.2003.05.013 12975461

[29] Ghesquieres H Cartron G Seymour JF Delfau-Larue MH Offner F Soubeyran P Clinical outcome of patients with follicular lymphoma receiving chemoimmunotherapy in the PRIMA study is not affected by FCGR3A and FCGR2A polymorphisms. Blood (2012) 120:2650–7. 10.1182/blood-2012-05-431825 22885164

[30] Racila E Link BK Weng WK Witzig TE Ansell S Maurer MJ A polymorphism in the complement component C1qA correlates with prolonged response following rituximab therapy of follicular lymphoma. Clin Cancer Res (2008) 14:6697–703. 10.1158/1078-0432.CCR-08-0745 PMC2907116 18927313

[31] Xu W Miao KR Zhu DX Fang C Zhu HY Dong HJ Enhancing the action of rituximab by adding fresh frozen plasma for the treatment of fludarabine refractory chronic lymphocytic leukemia. Int J Cancer (2011) 128:2192–201. 10.1002/ijc.25560 20635386

[32] Klepfish A Gilles L Ioannis K Rachmilewitz EA Schattner A Enhancing the action of rituximab in chronic lymphocytic leukemia by adding fresh frozen plasma: complement/rituximab interactions & clinical results in refractory CLL. Ann N Y Acad Sci (2009) 1173:865–73. 10.1111/j.1749-6632.2009.04803.x 19758239

[33] van Meerten T van Rijn RS Hol S Hagenbeek A Ebeling SB Complement-induced cell death by rituximab depends on CD20 expression level and acts complementary to antibody-dependent cellular cytotoxicity. Clin Cancer Res (2006) 12:4027–35. 10.1158/1078-0432.CCR-06-0066 16818702

[34] Boross P Jansen JH de Haij S Beurskens FJ van der Poel CE Bevaartj L The in vivo mechanism of action of CD20 monoclonal antibodies depends on local tumor burden. Haematologica (2011) 96:1822–30. 10.3324/haematol.2011.047159 PMC3232265 21880632

[35] Lee CH Romain G Yan W Watanabe M Charab W Todorova B IgG Fc domains that bind C1q but not effector Fcgamma receptors delineate the importance of complement-mediated effector functions. Nat Immunol (2017) 18:889–98. 10.1038/ni.3770 PMC6015732 28604720

[36] Montalvao F Garcia Z Celli S Breart B Deguine J Van Rooijen N The mechanism of anti-CD20-mediated B cell depletion revealed by intravital imaging. J Clin Invest (2013) 123:5098–103. 10.1172/JCI70972 PMC3859399 24177426

[37] Tobinai K Klein C Oya N Fingerle-Rowson G A Review of Obinutuzumab (GA101), a Novel Type II Anti-CD20 Monoclonal Antibody, for the Treatment of Patients with B-Cell Malignancies. Adv Ther (2017) 34:324–56. 10.1007/s12325-016-0451-1 PMC5331088 28004361

[38] Li Y Williams ME Cousar JB Pawluczkowycz AW Lindorfer MA Taylor RP Rituximab-CD20 complexes are shaved from Z138 mantle cell lymphoma cells in intravenous and subcutaneous SCID mouse models. J Immunol (2007) 179:4263–71. 10.4049/jimmunol.179.6.4263 17785867

[39] Dahal LN Huang CY Stopforth RJ Mead A Chan K Bowater JX Shaving Is an Epiphenomenon of Type I and II Anti-CD20-Mediated Phagocytosis, whereas Antigenic Modulation Limits Type I Monoclonal Antibody Efficacy. J Immunol (2018) 201:1211–21. 10.4049/jimmunol.1701122 PMC6082343 29997125

[40] Herter S Herting F Mundigl O Waldhauer I Weinzierl T Fauti T Preclinical activity of the type II CD20 antibody GA101 (obinutuzumab) compared with rituximab and ofatumumab in vitro and in xenograft models. Mol Cancer Ther (2013) 12:2031–42. 10.1158/1535-7163.MCT-12-1182 23873847

[41] Beurskens FJ Lindorfer MA Farooqui M Beum PV Engelberts P Mackus WJ Exhaustion of cytotoxic effector systems may limit monoclonal antibody-based immunotherapy in cancer patients. J Immunol (2012) 188:3532–41. 10.4049/jimmunol.1103693 PMC3311731 22368276

[42] Kennedy AD Beum PV Solga MD DiLillo DJ Lindorfer MA Hess CE Rituximab infusion promotes rapid complement depletion and acute CD20 loss in chronic lymphocytic leukemia. J Immunol (2004) 172:3280–8. 10.4049/jimmunol.172.5.3280 14978136

[43] Okroj M Eriksson I Osterborg A Blom AM Killing of CLL and NHL cells by rituximab and ofatumumab under limited availability of complement. Med Oncol (2013) 30:759. 10.1007/s12032-013-0759-5 24198205

[44] Horl S Banki Z Huber G Ejaz A Windisch D Muellauer B Reduction of complement factor H binding to CLL cells improves the induction of rituximab-mediated complement-dependent cytotoxicity. Leukemia (2013) 27:2200–8. 10.1038/leu.2013.169 PMC3826035 23760402

[45] Jilani I O’Brien S Manshuri T Jilani I O’Brien S Manshuri T Transient down-modulation of CD20 by rituximab in patients with chronic lymphocytic leukemia. Blood (2003) 102:3514–20. 10.1182/blood-2003-01-0055 12893761

[46] Stasilojc G Felberg A Urban A Kowalska D Ma S Blom AM Calcein release assay as a method for monitoring serum complement activity during monoclonal antibody therapy in patients with B-cell malignancies. J Immunol Methods (2020) 476:112675. 10.1016/j.jim.2019.112675 31629742

[47] Hallek M Cheson BD Catovsky D Caligaris-Cappio F Dighiero G Dohner H iwCLL guidelines for diagnosis, indications for treatment, response assessment, and supportive management of CLL. Blood (2018) 131:2745–60. 10.1182/blood-2017-09-806398 29540348

[48] Blom AM Volokhina EB Fransson V Stromberg P Berghard L Viktorelius M A novel method for direct measurement of complement convertases activity in human serum. Clin Exp Immunol (2014) 178:142–53. 10.1111/cei.12388 PMC4360204 24853370

[49] Uphoff CC Brauer S Grunicke D Gignac SM MacLeod RA Quentmeier H Sensitivity and specificity of five different mycoplasma detection assays. Leukemia (1992) 6:335–41. 1375305

[50] Blom AM Osterborg A Mollnes TE Okroj M Antibodies reactive to cleaved sites in complement proteins enable highly specific measurement of soluble markers of complement activation. Mol Immunol (2015) 66:164–70. 10.1016/j.molimm.2015.02.029 25795308

[51] Bordron A Bagacean C Mohr A Tempescul A Bendaoud B Deshayes S Resistance to complement activation, cell membrane hypersialylation and relapses in chronic lymphocytic leukemia patients treated with rituximab and chemotherapy. Oncotarget (2018) 9:31590–605. 10.18632/oncotarget.25657 PMC6114972 30167081

[52] Weng WK Levy R Expression of complement inhibitors CD46, CD55, and CD59 on tumor cells does not predict clinical outcome after rituximab treatment in follicular non-Hodgkin lymphoma. Blood (2001) 98:1352–7. 10.1182/blood.v98.5.1352 11520782

[53] Bannerji R Kitada S Flinn IW Pearson M Young D Reed JC Apoptotic-regulatory and complement-protecting protein expression in chronic lymphocytic leukemia: relationship to in vivo rituximab resistance. J Clin Oncol (2003) 21:1466–71. 10.1200/JCO.2003.06.012 12697868

[54] Middleton O Cosimo E Dobbin E McCaig AM Clarke C Brant AM Complement deficiencies limit CD20 monoclonal antibody treatment efficacy in CLL. Leukemia (2014) 29:107–14. 10.1038/leu.2014.146 24787488

[55] Ferriani VP Barbosa JE de Carvalho IF Complement haemolytic activity (classical and alternative pathways), C3, C4 and factor B titres in healthy children. Acta Paediatr (1999) 88:1062–6. 10.1080/08035259950168081 10565449

[56] da Silva KR Fraga TR Lucatelli JF Grumach AS Isaac L Skipping of exon 27 in C3 gene compromises TED domain and results in complete human C3 deficiency. Immunobiology (2016) 221:641–9. 10.1016/j.imbio.2016.01.005 26847111

[57] Olszewski WL Engeset A Haemolytic complement in peripheral lymph of normal men. Clin Exp Immunol (1978) 32:392–8. PMC1541330 99278

[58] Kaartinen M Kosunen TU Makela O Complement and immunoglobulin levels in the serum and thoracic duct lymph of the rat. Eur J Immunol (1973) 3:556–9. 10.1002/eji.1830030906 4128974

[59] Baig NA Taylor RP Lindorfer MA Church AK LaPlant BR Pettinger AM Induced resistance to ofatumumab-mediated cell clearance mechanisms, including complement-dependent cytotoxicity, in chronic lymphocytic leukemia. J Immunol (2014) 192:1620–9. 10.4049/jimmunol.1302954 PMC4391060 24431228

[60] Teeling JL French RR Cragg MS van den Brakel J Pluyter M Huang H Characterization of new human CD20 monoclonal antibodies with potent cytolytic activity against non-Hodgkin lymphomas. Blood (2004) 104:1793–800. 10.1182/blood-2004-01-0039 15172969

[61] Berinstein NL Grillo-Lopez AJ White CA Bence-Bruckler I Maloney D Czuczman M Association of serum Rituximab (IDEC-C2B8) concentration and anti-tumor response in the treatment of recurrent low-grade or follicular non-Hodgkin’s lymphoma. Ann Oncol (1998) 9:995–1001. 10.1023/A:1008416911099 9818074

[62] Taylor RP Lindorfer MA Analyses of CD20 Monoclonal Antibody-Mediated Tumor Cell Killing Mechanisms: Rational Design of Dosing Strategies. Mol Pharmacol (2014) 86:485–91. 10.1124/mol.114.092684 PMC4201137 24944188

[63] Taylor RP Lindorfer MA Fcgamma-receptor-mediated trogocytosis impacts mAb-based therapies: historical precedence and recent developments. Blood (2015) 125:762–6. 10.1182/blood-2014-10-569244 25498911

[64] Vaughan AT Chan CH Klein C Glennie MJ Beers SA Cragg MS Activatory and inhibitory Fcgamma receptors augment rituximab-mediated internalization of CD20 independent of signaling via the cytoplasmic domain. J Biol Chem (2015) 290:5424–37. 10.1074/jbc.M114.593806 PMC4342459 25568316

[65] Hekman A Honselaar A Vuist WM Sein JJ Rodenhuis S ten Bokkel Huinink WW Initial experience with treatment of human B cell lymphoma with anti-CD19 monoclonal antibody. Cancer Immunol Immunother (1991) 32:364–72. 10.1007/BF01741331 PMC11038497 1706642

