# Supplementary material for: Monitoring of the Complement System Status in Patients With B-Cell Malignancies Treated With Rituximab
Source: Front Immunol. 2020 Nov 19;11:584509. doi: 10.3389/fimmu.2020.584509 (PMC7710700; doi:10.3389/fimmu.2020.584509)
Supplement: Supplementary file 1 [file Table_1.docx]

Supplementary Table 1

List of patients, who significantly decreased CDC activity in their post-infusion serum samples

* - p < 0.05, *** - p<0.001, according to Sidak’s multiple comparison test. All blood samples were collected immediately before and after each rituximab infusions.

grey shedding – no infusion / missing sample

| **Patient #** | **infusion** | | | | | | |
| --- | --- | --- | --- | --- | --- | --- | --- |
|  | **2nd** | **3rd** | **4th** | **5th** | **6th** | **7th** | **8th** |
| 1 |  |  |  |  |  |  |  |
| 6 |  |  |  |  |  |  |  |
| 8 |  |  |  |  |  |  |  |
| 9 |  |  |  | * |  |  |  |
| 10 |  |  |  |  |  |  |  |
| 11 |  |  |  |  |  |  |  |
| 12 |  |  |  |  |  |  |  |
| 19 |  |  |  |  |  |  |  |
| 20 |  |  |  |  |  |  |  |
| 31 |  |  |  |  |  |  |  |
| 17 |  |  |  |  |  |  |  |
| 18 | *** |  |  |  |  |  |  |
| 21 |  |  |  |  |  |  |  |
| 23 |  |  |  |  |  |  |  |
| 26 |  |  |  |  |  |  |  |
| 27 |  |  |  |  |  |  |  |
| 33 |  |  |  |  |  |  |  |

Supplementary Table 2

List of CLL patients, who significantly increases C4d levels (top section) and TCC (bottom section) in their serum samples after administration of rituximab

** - p < 0.01, *** - p<0.001, according to Sidak’s multiple comparison test

Grey shedding – missing sample

| **Patient #** | **Infusion** | | | |
| --- | --- | --- | --- | --- |
|  | **1st** | **2nd** | **3rd** | **4th** |
| 17 | ** |  |  |  |
| 18 | *** | *** |  |  |
| 21 | *** |  |  |  |
| 23 | *** |  |  |  |
| 26 | *** |  |  |  |
| 27 |  |  |  |  |
| 33 | *** | ** | *** | *** |

| **Patient #** | **Infusion** | | | |
| --- | --- | --- | --- | --- |
|  | **1st** | **2nd** | **3rd** | **4th** |
| 17 | *** |  | *** |  |
| 18 | *** |  |  |  |
| 21 |  |  |  |  |
| 23 |  |  |  |  |
| 26 | *** |  |  |  |
| 27 |  |  |  |  |
| 33 | *** | *** |  |  |
